# The Diagnosis and Treatment of Barbers' Rashes

**Published:** 1907-09-28

**Authors:** 


### Dermatology.

### THE DIAGNOSIS AND TREATMENT OF BARBERS' RASHES.

Not every eruption which appears upon the beard region after a visit to a hairdresser is "barbers' itch," or a "barber's rash." These terms were originally applied to true ringworm of the beard only, but they have since become elastic enough to include several different types of coccigenic, impetiginous, and eczematous affections which happen to be first noticed within a period of time, which may be most variable, after the application of the barber's tools. The question of *post hoc* or *propter hoc* is one that is sometimes very difficult for the practitioner to decide, especially when he remembers that his diagnosis may cause legal proceedings to be taken by the patient against the, perhaps, perfectly innocent barber. Not only is it notoriously difficult to prove wilful negligence in such cases, but the doctor may be asked some extremely awkward questions in the witness-box. The ordinary white coccus inhabiting the normal human epidermis has been known, under altered conditions of soil, to acquire a sudden virulence, and to produce lesions indistinguishable from those caused by pyogenic organisms. It is not wise, therefore, to pronounce hastily with regard to the connection of the skin trouble with a visit to a barber.

### Ringworm of the Beard.

There are several characteristics which serve to distinguish *tinea barbæ* from other eruptions of this region. They are (*a*) small areas of scaliness surrounding the hair follicles; (*b*) the presence of dull-looking, broken, or thickened hairs, which prove to be brittle on attempting to epilate them; (*c*) scattered nodules and reddened, infiltrated lumps which may proceed to suppuration. These lumps have considerable diagnostic value, as they are not seen in coccigenic sycosis. If the patient has shaved since finding out the places circinate areas, with slightly raised margins, may be plainly visible. Scrapings from these edges mounted in a drop of potash solution, together with any suspicious hair, should reveal the presence of spores or mycelium when examined under the microscope with a one-sixth objective. The writer well remembers one case in which lumps were present, together with some crust formation, but the patient also had similar areas upon the upper arm. In this case no mycelium was found, there were no thickened hairs, and the whole trouble cleared up rapidly under potassium iodide internally. Tertiary syphilis had simulated ringworm remarkably well! The hair of the affected parts should be clipped quite close, and an ointment containing 10 grains of nitrate of mercury and ½ dr. of carbolic acid to the ounce of lanoline may be rubbed in night and morning. Epilation of any diseased hairs should also be practised, as far as possible. A lotion of dilute cyllin, or perchloride of mercury (1 in 5,000) is useful for bathing the parts.

### Sycosis and Folliculitis.

These two affections are practically identical—*i.e.* they consist in a primary infection of the hair-follicle with streptococci. There is, perhaps, rather more pus formation in this the coccigenic variety of sycosis (ringworm of the beard being sometimes known as hyphogenic sycosis), and lumpy infiltration is not a marked feature. The hairs may frequently be seen emerging from the centre of an infected follicle, and they offer some resistance to epilation. Crusting is common, and this symptom causes the condition to be mistaken for simple impetigo, which does not necessarily commence in the hair-follicles. The infective process may spread to adjacent parts, and boils may occur upon the neck and face. Antiseptic lotions such as the above-mentioned should be applied warm two or three times a day, and an ointment containing 5 grs. of the red sulphide of mercury and 10 minims of ichthyol in 1 oz. of lanoline or vaseline may be applied afterwards, the hair being kept short as before. In simple impetigo the official ammoniated mercury ointment is one of the most useful salves.

An irritative form of papular eczema is sometimes seen after shaving, whether this be carried out at home or at a barber's. The quality of the soap employed for preparing the lather is popularly held responsible, and very numerous are the patent shaving-soaps advertised which are warranted to give "an easy shave." While admitting that a pure soap should be used for this purpose, it is equally important to secure a thorough sterilisation of the razor and also of the shaving-brush immediately before use. A little cold cream containing some oleo-palmitate of zinc is both pleasant and serviceable as a toilet application just after a shave when such an irritation threatens to appear. Dusting-powders containing starch are useful cosmetically, but boracic acid is best avoided by those with sensitive skins.

